# No Impact of Long-Term Fingolimod Treatment on Fecal Secretory Immunoglobulin A Levels in Patients With Multiple Sclerosis

**DOI:** 10.3389/fcell.2020.567659

**Published:** 2020-09-25

**Authors:** Hernan Inojosa, Judith Eisele, Undine Proschmann, Sebastian Zeissig, Katja Akgün, Tjalf Ziemssen

**Affiliations:** ^1^Department of Neurology, Multiple Sclerosis Center, Center of Clinical Neuroscience, University Hospital Carl Gustav Carus, Dresden University of Technology, Dresden, Germany; ^2^Department of Medicine I, University Hospital Carl Gustav Carus, Dresden University of Technology, Dresden, Germany

**Keywords:** multiple sclerosis, fingolimod, secretory immunglobulin A, sIgA, microbiome, gut immune system

## Abstract

**Background:**

Fingolimod (FTY) is a sphingosine 1 phosphate (S1P) agonist with significant effects on immune cell distribution used as an effective disease modifying therapy in multiple sclerosis (MS) patients. Animal studies have demonstrated that a dysregulation of egress of murine secretory Immunglobulin A (sIgA)+ plasmablasts from Peyer’s patches in FTY-treated mice reduced fecal sIgA levels. Alterations in intestinal levels of sIgA could modify the gut microbiome and homeostasis in humans. We analyzed the effect of FTY on the fecal and salivary sIgA levels as marker of the humoral immune system in the gut.

**Methods:**

Twenty five people with confirmed MS diagnosis according to 2010 revised McDonald’s criteria and on long-term continuous treatment at the MS Center in Dresden, Germany were enrolled in this exploratory cross-sectional study. Fecal and salivary sIgA were analyzed after at least 12 months of treatment with FTY or Glatiramer acetate (GA).

**Results:**

Fifteen MS patients on FTY and 10 on GA participated in this study. The mean fecal sIgA concentration of both groups was not decreased compared to reference values and did not demonstrate significant differences between them (FTY 3323.13 μg/g +/− 2094.72; GA 2040.65 μg/g +/− 1709.07). A similar pattern was seen in the salivary sIgA and serum immunoglobulins levels.

**Conclusion:**

In this pilot study, we could not confirm the decrease of fecal sIgA after a long-term treatment with FTY. Further longitudinal studies should evaluate the effects of MS treatments on the gut immune system in more detail.

## Introduction

Multiple sclerosis (MS) is a chronic inflammatory disease of the central nervous system (CNS) induced by an imbalance of the immune-regulatory network. Disease-modifying treatments (DMTs) have shown high efficacy altering the disease course through immunomodulatory mechanisms decreasing inflammation at the CNS (Brück et al., 2013). Fingolimod (FTY), the first oral approved DMT for treatment of highly active forms of relapsing-remitting MS, is a sphingosine-1-phosphate (S1P) receptor modulator that regulates S1P-mediated lymphocyte trafficking from lymphoid tissues, impairing therefore peripheral lymphocyte recirculation (Brinkmann et al., 2010; Thomas et al., 2017a). Thus, fingolimod leads to a rapid reduction of absolute lymphocyte count in peripheral blood in addition to modulatory effects on antigen-presenting cells that mediate a rebalancing of auto-reactive immune responses in MS patients (Thomas et al., 2017b; Kaufmann et al., 2018; Sehr et al., 2020). Additionally, the effect of this drug on the different S1P-receptors is non-selective. While the S1P receptors S1P1, S1P2 and S1P3 are ubiquitously expressed, the subtypes S1P4 and S1P5 are restricted to certain cell types (Blaho and Hla, 2014).

In animal models, FTY treatment delayed the bacterial clearance when mice were infected with luciferase-tagged *C. rodentium* (Murphy et al., 2012). FTY-treated *C. rodentium*-infected mice had enhanced colonic inflammation, higher colon mass, colon histopathology and neutrophil infiltration than vehicle-treated infected animals. In addition, they had significantly lower numbers of colonic dendritic cells, macrophages and T cells, leading to an impaired local immune response in the colon. Animal studies demonstrated as well that the emigration of immunoglobulin A (IgA)-secreting plasma cell precursors was controlled by S1P receptor expression during B cell differentiation in the Peyer’s patches (PPs) (Gohda et al., 2008; Kleinwort et al., 2018), which could be shown in S1P4-deficient mice as well as in mice with FTY treatment (Kunisawa et al., 2007). Functional antagonism of S1P-mediated signaling by FTY treatment selectively accumulated IgA+ plasmablasts in lymphatic regions of PPs and impaired the production of specific fecal IgA responses (Gohda et al., 2008).

These findings suggest a relevant S1P signaling modulation of B cell function by FTY, reflected by a reduction of the plasmablast population in the intestinal lamina propria and resulting in an impairment of antigen-specific sIgA production against orally administered antigens (Kunisawa et al., 2007; Kleinwort et al., 2018).

Additionally, a disruption in sIgA production could drive to an impairment in the gut immune function with further consequences for several inflammatory diseases, such as e.g., the MS (Okai et al., 2016; Salas-Cuestas et al., 2017; Morshedi et al., 2019; Mirza et al., 2020; Reynders et al., 2020). There is a close relationship between IgA and colitogenic bacteria that increase the risk for intestinal diseases, as evidence shows that this immunoglobulin coats several of these bacteria (Palm et al., 2014; Rojas et al., 2019). Interestingly, a study could demonstrate a reduction in IgA-coated fecal bacteria in MS patients during acute relapses compared to patients on remission, suggesting a role of IgA in the regulation of neuroinflammation (Rojas et al., 2019). The relationship between the sIgA, the microbiome and the disease activity is an interesting and promising field for a further characterization of people with MS.

Due to the potential impact of FTY on intestinal B cell activity and function, an analysis in human patients could depict a possible sIgA-decrease mediated by this DMT, which was already demonstrated in mice. Therefore, the aim of this small pilot study was to evaluate the effects of a long-term treatment with FTY in fecal sIgA levels in people with MS. In parallel, salivary sIgA was analyzed in this group of patients as there is evidence of expression of S1P receptors (specifically type 1) on salivary gland cells (Sekiguchi et al., 2008; Li et al., 2016). Analyzing the potential effects of the immunomodulatory therapies used in MS in the gut immune system could support the understanding of the MS disease course.

## Methods

We performed an observational study in a real world cohort of patients with MS diagnosis according to 2010 revised McDonald’s criteria (Polman et al., 2011). Patients with relapsing remitting disease subtype, older than 18 years and under treatment with fingolimod for at least 12 months were included. A reference group of patients with glatiramer acetate (GA) treatment was also included. Patients with potential gastrointestinal disease were excluded by careful examination and anamnesis. The study was approved by the institutional review board of the University Hospital of Dresden and patients gave a written informed consent. To increase the robustness of the sIgA measurements, two measures were performed for each patient at two different days within 3 months.

Standard blood testing was performed for routine blood parameters at the Institute of Clinical Chemistry and Laboratory Medicine, University Hospital in Dresden, Germany (Kaufmann et al., 2018) in accordance to standards required by DIN-EN-ISO-15189:2014 for medical laboratories. Whole blood samples were collected in ethylene diamine tetra acetic acid (EDTA) for processing. Blood testing included complete blood cell count and serum immunoglobulins IgA, IgM, and IgG.

Fecal and salivary samples were collected at the MS Center in Dresden between 8:00 and 12:00 in the morning. The method used for saline extraction of immunoglobulins was adapted from the “IDK^®^ sIgA ELISA” manual (Immundiagnostik AG, Behnsheim, Germany). A wash buffer concentrate was used in the preparation of the samples (WASHBUF). No food or liquid was consumed at least 1 h before sample collection. Saliva samples were centrifuged at 3000 g for 10 min before 1:2000 dilution in wash buffer. 100 μl of this final dilution were used for the analysis.

Fecal samples were standardized with the following preparation methods: first, stool samples were thawed with mechanical homogenization and then diluted 1:100 using an extraction buffer (1:2.5 diluted *IDK Extract*^®^). For this first dilution a standard measure of 15 mg of each sample was used and an homogenous suspension was obtained. Afterward, the supernatant of this procedure was further diluted 1:125 in wash buffer. Hundred microliter of this final dilution were used for the analysis.

Samples were analyzed for sIgA using a modification of a commercially available enzyme-linked immunosorbent assays (ELISA) (Immundiagnostik AG, Behnsheim, Germany). Free sIgA in the supernant was measured in the analyzed samples. Incubations and rinsing were done according to the manufacturer’s guidelines in a sealed humidified chamber. Absorbance was read immediately after the assays by spectrophotometry at 450 nm. Lower limits of normal range were 102 μg/ml and 510 μg/ml for saliva and fecal sIgA, respectively.

## Statistical Analysis

Normal distribution of data was visually assessed using quantile-quantile plots and confirmed with Shapiro–Wilk tests. Quantitative population characteristics were presented as measures of central tendency (mean, median), followed by standard deviation (SD) or interquartile range (IQR). A descriptive specification of (crude) mean values and standard deviations occurred. Differences in fecal sIgA and serum IgA concentrations between the treatment groups were assessed with unpaired *T*-tests. All statistical analyses were performed using IBM SPSS version 25.0 (IBM Corporation, Armonk, NY, United States).

## Results

A group of 25 MS patients participated in this study, among which 15 were treated with FTY and 10 with GA (Table 1). After 20.87 months (*SD* = 7.59) of treatment, fecal sIgA was not decreased in the FTY-treated patients in relationship to reference values. No significant difference was found in the fecal sIgA between the FTY group and the GA group [*t*(23) = 1.5, *p* = 0.411] at the time of evaluation. A similar pattern was seen in the salivary sIgA, where no difference was detectable between the groups (*p* = 0.748).

**TABLE 1:** Secretory and serum IgA in patients treated with FTY and GA.

	FTY (*n* = 15)	GA (*n* = 10)	*p*
Females (*n*, %)	5 (33.3%)	10 (100%)	*p* < 0.05
Age in years (mean, *SD*)	43.67 (*SD* = 7.98)	46.40 (*SD* = 10.71)	*p* = 0.115
EDSS (median, IQR)	3.0 (2.0–4.0)	2.0 (2.0–3.0)	*p* = 0.327
Fecal sIgA in μg/g feces (mean, SD)	3323.13 (*SD* = 2094.72)	2040.65 (*SD* = 1709.07)	*p* = 0.411
Saliva sIgA in μg/ml (median, IQR)	667.03 (*SD* = 359.72)	778.85 (*SD* = 288.67)	*p* = 0.748
Leukocytes in GPt/l (mean, *SD*)	4.20 (*SD* = 1.41)	6.13 (*SD* = 1.50)	*p* = 0.880
Lymphocytes in GPt/l (mean, *SD*)	0.42 (*SD* = 0.17)	1.78 (*SD* = 0.58)	*p* < 0.05
Serum IgA in g/l (mean, *SD*)	1.78 (*SD* = 0.91)	1.78 (*SD* = 0.78)	*p* = 0.412
Serum IgM in g/l (mean, *SD*)	0.74 (*SD* = 0.26)	1.25 (*SD* = 0.58)	*p* = 0.255
Serum IgG in g/l (mean, *SD*)	8.54 (*SD* = 1.69)	11.58 (*SD* = 3.12)	*p* = 0.269

Similarly, assessing serum immunoglobulins - IgA, IgM, and IgG, there was no statistically significant difference between the FTY- and GA-treated patients. FTY-treated patients showed furthermore the targeted therapeutic decrease in the absolute lymphocyte counts with lower levels compared to the GA-treated patients (0.42 GPt/l, *SD* = 0.76, *p* < 0.05).

## Discussion

In this small exploratory study, we assessed fecal sIgA in a human population of MS patients on different DMTs.

The sIgA response belongs to the largest humoral immune system and serves as the first line of defense in protecting the intestinal epithelium from enteric toxins and pathogenic microorganisms (Brandtzaeg, 2009; Brandtzaeg, 2013). The IgA is a polymeric immunoglobulin with a “secretory component” produced by plasmablasts in the intestinal mucosa after B cell induction in lymphoid tissue (Brandtzaeg, 2013). Through a process known as immune exclusion, sIgA promotes the clearance of antigens and pathogenic microorganisms from the intestinal lumen by blocking their access to epithelial receptors, entrapping them in mucus and facilitating their removal by peristaltic and mucociliary activity (Brandtzaeg, 2009). Additionally, the sIgA may have the capacity to directly regulate bacterial virulence factors accounting for protective effects against several antigens and to transport immune complexes across the intestinal epithelium to lymphoid tissues with a regulation of pro-inflammatory responses (Mantis et al., 2002, 2011; Boullier et al., 2009; Chairatana and Nolan, 2017). In addition, sIgA modulates the mucosal immunity and intestinal homeostasis through mechanisms that have only recently been revealed (Brandtzaeg, 2013). The sIgA influences also the gut microbiota in a complex relationship through Fab-dependent and -independent mechanisms, an effect that is already present on neonatal exposure to commensal microorganisms (Corthesy, 2007; Sekirov et al., 2010; Mantis et al., 2011; Schofield and Palm, 2018; Chang and Kao, 2019).

In our study, we could not demonstrate a decrease in the fecal sIgA levels in a group of people with MS after a long-term treatment with FTY. We based our hypothesis on previously reported animal studies, where the treatment with FTY or the inhibition of the S1P receptor occasioned a decrease in sIgA levels in stool as result of an impairment of lymphocytes trafficking into the gut mucosa and PPs (Kunisawa et al., 2007; Gohda et al., 2008; Boullier et al., 2009; Murphy et al., 2012; Kleinwort et al., 2018).

These results should be carefully interpreted considering that they were obtained on a setting of a cross-sectional design, with an unequal gender distribution due to a random non-statistical sampling method. Additionally, levels of free sIgA in the supernant were measured in the selected assay. It should be considered that a great part of the fecal sIgA is bound to commensal bacteria as it coats both aerobic and anaerobic bacteria in healthy controls (van der Waaij et al., 1996; Hoces et al., 2020).

The analysis of the intestinal immunity and microbiome of people with MS is a promising field that could aid in understanding the pathophysiology of the disease (Mirza et al., 2020). The study of DMTs-derived adverse events is furthermore crucial for the follow-up of MS patients. Although our pilot study did not reveal differences with GA-treated patients regarding fecal and saliva sIgA, FTY as well as other DMTs could have an effect in the gut humoral immune system, affecting consequently the gut microbiome. This effect would not only be relevant in MS patients, but also in other inflammatory diseases such as the inflammatory bowel disease or irritable bowel syndrome (Petta et al., 2018; Liu et al., 2020). This is of great importance as the blockade of the S1P/S1PR1 axis is emerging as a new therapeutic approach to control the aberrant leukocyte migration into the mucosa in inflammatory bowel disease (Danese et al., 2018).

However, two important limitations should be considered in our exploratory study. First, we assessed patients in a small cross-sectional exploratory study. Here, we had a small participating group with unequal characteristics. A further larger longitudinal evaluation with a more controlled sampling and an assessment of sIgA levels before and after FTY treatment should be performed to validate our results. Due to the small group size, a power analysis below 80% was obtained in this study, with a probability of type II errors to occur. Additionally, the gender distribution (as well as other characteristics of the patients) should be controlled as in this pilot study it was unequal between the treatment groups and may be a confounding factor. Second, we had an uncontrolled setting regarding patient’s commensal microbiome, inflammatory markers or alimentation. A parallel study of gut microbiome, IgA-coating microorganisms, as well as serum inflammatory markers would be helpful for further characterization of the patients and interpretation of our results.

As newer therapeutic options are available for MS patients, further controlled prospective studies involving larger patient cohorts should be performed to evaluate the effect of DMTs (e.g., Fingolimod, B cell depleting agents as Ocrelizumab) on the immune function of people with MS and in the gut microbiome.

## Data Availability Statement

The raw data supporting the conclusions of this article will be made available by the authors, without undue reservation.

## Ethics Statement

The studies involving human participants were reviewed and approved by the Ethics Commitee of the Technical University Dresden. The patients/participants provided their written informed consent to participate in this study.

## Author Contributions

HI, KA, and TZ: study concept and design. JE: acquisition of data. HI, UP, SZ, JE, and KA: analysis and interpretation of data. HI, JE, KA, and TZ: drafting of the manuscript. SZ, UP, KA, and TZ: critical revision of the manuscript for important intellectual content. HI and JE: statistical analysis. All authors contributed to the article and approved the submitted version.

## Conflict of Interest

UP received speaker fee from Merck, Biogen, and Bayer and received additionally personal compensation from Biogen and Roche for consulting service. SZ received personal compensation from Abbvie, Amgen, Biogen, Bristol-Myers Squibb, Celltrion, Falk, Ferring, Mylan, Janssen-Cilag, MSD, Roche, Pfizer, Takeda for consulting services. KA received personal compensation for from Biogen Idec, Roche, Merck, and Sanofi for consulting service. TZ received personal compensation from Biogen Idec, Bayer, Novartis, Sanofi, Teva, and Synthon for consulting services and received additional financial support for research activities from Bayer, Biogen Idec, Novartis, Teva, and Sanofi Aventis. The remaining authors declare that the research was conducted in the absence of any commercial or financial relationships that could be construed as a potential conflict of interest.
